# Transcriptome profile of OVCAR3 cisplatin-resistant ovarian cancer cell line

**DOI:** 10.1186/1471-2105-15-S10-P21

**Published:** 2014-09-29

**Authors:** Shruti S Sakhare, Gautam G Rao, Sammed N Mandape, Siddharth Pratap

**Affiliations:** 1 Bioinformatics Core, Meharry Medical College, Nashville, TN, 37208, USA; 2 Obstetrics, Gynecology and Reproductive Sciences, University of Maryland, Baltimore, MD, 21201, USA

## Background

The NIH:OVCAR-3 is a cisplatin refractory cell line established from malignant ascites of a patient with progressive adenocarcinoma of the ovary after combination chemotherapy with cyclophosphamide, Adriamycin, and cisplatin [1]. Thus, OVCAR3 serves as a model cell line for drug resistance in ovarian cancer. Here, we perform a comparative transcriptome analysis from the US National Cancer Institute human tumor cell line anticancer drug screen (NCI60) dataset [2]. Our results indicate a specific gene transcription profile of OVCAR3 genes relative to non-cancerous Human Ovarian Surface Epithelial cells (HOSE) and drug sensitive Serous Ovarian Cancer Epithelial Samples (CEPI) and SKOV3 cell lines. Pathway enrichment analysis from OVCAR3 unique transcripts was conducted using KEGG; Disease and Drug term enrichment used the PharmGKB [3] databases.

## Materials and methods

Datasets from the NCI60 were obtained from the Gene Expression Omnibus (GEO) of NCBI [4] (OVCAR3 and SKOV3 from series GSE2003, OSE and CEPI from series GDS3592 and GSE14407). Transcriptome data analysis was conducted with Partek Genomics Suite version 6.6. The WEB-based GEne SeT AnaLysis Toolkit (WebGestalt) was used to perform enrichment analysis [5]. Genes present in KEGG pathway, PharmGKB Disease, and Drug terms enrichment sets were connected and expanded to one degree of biological interaction using the Michigan Molecular Interactions databases plugin [6] and visualized using Cytoscape version 2.8.3 [7].

## Results

Transcriptome analysis of OVCAR3 specific gene expression changes resulted in 160 significant transcripts with a fold change > ±2 and an ANOVA derived Benjamini Hochberg adjusted p-value < 0.001. Enrichment analysis using a Hypergeometric test identified 189 PharmGKB Disease terms, 90 Drug terms and 31 KEGG pathways associated with these genes. A union of the disease, drug and KEGG pathway gene lists yielded 14 common genes for the dataset which were unique to OVCAR3 cells versus SKOV3 and CEPI (Table 1).

**Table 1:** Transcripts differentially expressed in OVCAR3 versus SKOV3 and CEPI having significant enrichment scores in KEGG Pathway, PharmGKB Drug and Disease databases.

Gene Symbol	Gene Name	Ensembl
ABCC4	ATP-binding cassette, sub-family C (CFTR/MRP), member 4	ENSG00000125257
AKR1C2	aldo-keto reductase family 1, member C2 (dihydrodiol dehydrogenase 2; bile acid binding protein; 3-alpha hydroxysteroid dehydrogenase, type III)	ENSG00000151632
MLH1	mutL homolog 1, colon cancer, nonpolyposis type 2 (E. coli)	ENSG00000076242
GLS	glutaminase	ENSG00000115419
ITGA3	integrin, alpha 3 (antigen CD49C, alpha 3 subunit of VLA-3 receptor)	ENSG00000005884
UGT1A6	UDP glucuronosyltransferase 1 family, polypeptide A6	ENSG00000167165
PPARG	peroxisome proliferator-activated receptor gamma	ENSG00000132170
NNMT	nicotinamide N-methyltransferase	ENSG00000166741
PTGIS	prostaglandin I2 (prostacyclin) synthase	ENSG00000124212
ABCC3	ATP-binding cassette, sub-family C (CFTR/MRP), member 3	ENSG00000108846
UGT1A1	UDP glucuronosyltransferase 1 family, polypeptide A1	ENSG00000241635
ERBB2	v-erb-b2 erythroblastic leukemia viral oncogene homolog 2, neuro/glioblastoma derived oncogene homolog (avian)	ENSG00000141736
AKR1C1	aldo-keto reductase family 1, member C1 (dihydrodiol dehydrogenase 1; 20-alpha (3-alpha)-hydroxysteroid dehydrogenase)	ENSG00000187134
FGF2	fibroblast growth factor 2 (basic)	ENSG00000138685

## Conclusions

**Figure 1:**
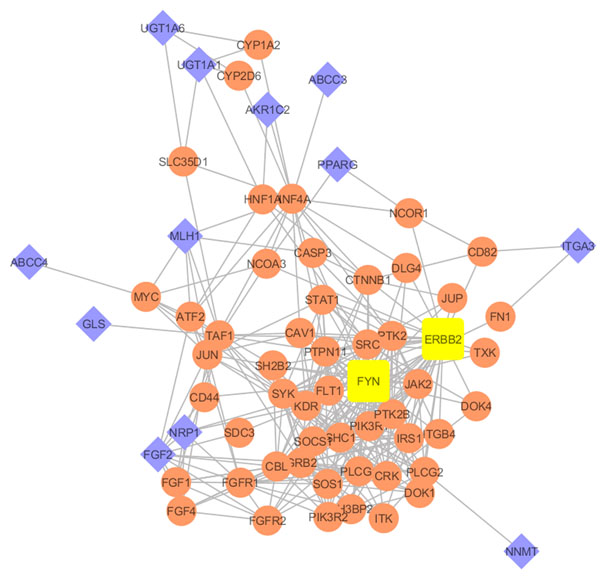
Interaction network of OVCAR3 unique genes. Diamond and rectangle nodes are seed nodes of 14 OVCAR3 unique genes; circular nodes are 1 degree of biological interactions; rounded rectangular nodes are the highly connected hubs in the network (FYN and ERBB2).

This list of OVCAR3 unique genes, and the resulting interactions graph (Figure 1) represent potential pathways of drug resistance associated genes in ovarian cancer. Notably, ERBB2 (HER2) and FYN are the hub genes of the interaction network specific for OVCAR3 cell line. Thus, they may provide valuable insights into the drug resistance etiology of ovarian cancer. ERBB2 (HER2) has previously been reported to interact with Estrogen Receptor (ESR2) in Breast Cancer [8] and FYN has been implicated in Glioblastoma and T-cell Lymphomas [9,10]; however their detailed roles in Ovarian Cancer have only recently been studied, warranting further investigation [11,12].
